# Subclinical endometritis and postpartum ovarian resumption in respect to TNF-α, IL-8 and CRP in Egyptian buffaloes

**DOI:** 10.21451/1984-3143-AR2019-0027

**Published:** 2020-01-27

**Authors:** Doaa H. Elsayed, Fakhri E. El-Azzazi, Yasmina K. Mahmoud, Sherif M. Dessouki, Eman A. Ahmed

**Affiliations:** 1 Department of Theriogenology, Faculty of Veterinary Medicine, Suez Canal University, Ismailia, Egypt; 2 Department of Animal Production, Faculty of Agriculture, Suez Canal University, Ismailia, Egypt; 3 Department of Biochemistry, Faculty of Veterinary Medicine, Suez Canal University, Ismailia, Egypt; 4 Department of Animal Production, Faculty of Agriculture, Cairo University, Giza, Egypt; 5 Department of Pharmacology, Faculty of Veterinary Medicine, Suez Canal University, Ismailia, Egypt

**Keywords:** buffaloes, CRP, cytokines, ovarian cyclicity, subclinical endometritis

## Abstract

The study was carried out to find the relation between subclinical endometritis (SCE) and postpartum (pp.) ovarian resumption as well as to evaluate serum and endometrial TNF-α, IL-8 and serum CRP in buffaloes with and without SCE. Thirty-nine pluriparous buffaloes at the 3^rd^ (W3), 5^th^ (W5) and 7^th^ (W7) week pp. were involved in this experiment. The parity of the buffaloes ranged from 4 to 8 with an average 5.8±0.2. Subclinical endometritis was diagnosed by the percentage of polymorphonuclear cells (PMNs) in uterine cytology obtained from endometrial cytobrush at W5 and W7. The cut-off point of PMNs% in buffaloes for SCE was ≥ 6% at W5 or ≥ 4% at W7. According to PMNs%, buffaloes were divided into SCE group (n=27) and non-SCE group (n=12). Ovarian cyclicity was monitored by rectal palpation, ultrasonography and progesterone assay at W3, W5 and W7. Serum and endometrial TNF-α, IL-8 and serum CRP were estimated at W5 and W7. Buffaloes with SCE (55.6%) showed delayed ovarian activity as compared to non-SCE (16.7%) animals (*P*=0.036). Significant increase in serum cytokines and CRP levels were detected at W5 (*P* ˂0.05) and W7 (*P* <0.01) in SCE buffaloes as compared to non-SCE. Endometrial levels of cytokines were significantly (*P* ˂0.05) elevated in SCE buffaloes. Serum and endometrial cytokines showed significant positive correlation. Furthermore, levels of TNF-α, IL-8 and CRP exhibited significant positive correlation with PMNs%. In conclusion, SCE delayed postpartum ovarian cyclicity in buffaloes. Moreover, TNF-α, IL-8 and CRP assessments could be efficient tools in prediction of SCE in buffaloes.

## Introduction

Identification and recognition of biomarkers help in early diagnosis and assessment of the animal’s pathophysiological condition of some diseases (Manimaran et al., 2016). Among these diseases is, asymptomatic endometritis known as subclinical endometritis (SCE) or cytological endometritis (Boby et al., 2017; Ghanem et al., 2015). Buffaloes suffered from SCE showed increased days to first estrus, prolonged days open (Ali et al., 2009), repeat breeding, reduced conception and pregnancy rates (Senosy and Hussein, 2013). Therefore, these impairments in fertility (Singh et al., 2018) as well as reproductive performance gave rise to threats of culling and economic losses (Pande et al., 2013).

Uterine infection altered endometrial and postpartum (pp.) ovarian functions (Nehru et al., 2019) that subsequently prolonged inter-calving intervals in buffaloes (Hanafi et al., 2008). Moreover, infertility produced from ovarian dysfunction is more pronounced among buffaloes than cows (Elwishy et al., 1971). Previous studies demonstrated higher incidence of pp. uterine infection in Egyptian buffaloes than cows (Azawi, 2008). Therefore, buffalos' breeders encounter problems of reduced reproductive pattern and prolonged inter-calving intervals (Boby et al., 2017; Sah and Nakao, 2006).

Impairment of peri-partum immune function is a causal factor for the progress of pp. uterine infections in bovines (Bhadaniya et al., 2019; Ghanem et al., 2002; Patra et al., 2013). Innate immune system is involved in pathogen recognition in bovine endometritis and reproductive failure. Its action is mainly achieved through toll-like receptors (TLRs) induction that stimulate adaptive immune response by pro-inflammatory cytokines elaboration (Ajevar et al., 2014). Clear examples for such pro-inflammatory cytokines are tumor necrosis factor alpha (TNF-α) and interleukins (IL-1, IL-6 and IL-8) (Boby et al., 2017; Manimaran et al., 2016; Wang et al., 2018) that have a fundamental role in the intracellular communication among uterine cells (Loyi et al., 2013). Although cytokines have major roles in steroidogenesis and ovulation process, their overproduction impair these processes (Stassi et al., 2017). Tumor necrosis factor alpha, multifunctional pro-inflammatory cytokine, secreted by many cells as macrophages, T lymphocytes and ovarian granulosa cells (Price and Sheldon, 2013). Interleukine-8, the primary neutrophil chemokine, is produced by activated leukocytes that enhance the targeted release of neutrophils and T lymphocytes in order to eliminate pathogens in bovine uterus (Bhadaniya et al., 2019; Mossallam et al., 2015). Both TNF-α and IL-8 are essential in uterine immune cascade of SCE buffaloes via their upregulation in the endometrium (Loyi et al., 2013). Therefore, they could be useful for accurate and early diagnosis of SCE than only determining of polymorphonuclear cells % (PMNs) in buffaloes (Loyi et al., 2013).

Pro-inflammatory cytokines from monocytes and macrophages are effective stimulators for the production of acute phase proteins (APPs) such as C-reactive protein (CRP), haptoglobin and serum amyloid A (Kaya et al., 2016; Manimaran et al., 2016). Acute phase proteins are synthesized in liver and their levels in serum increase through the first few weeks after calving, in response to uterine infection (Brodzki et al., 2015; Tóthová et al., 2008). Upregulation of APPs as well as PMNs are key features of uterine inflammation that could be beneficial for successful recovery of inflammation, healthy uterine involution and in turn high conception rate (Chapwanya et al., 2009). C-reactive protein, non-glycosylated protein, is an important element of the innate defense system (Du Clos and Mold, 2001) that produced from liver toward infection and stressful condition (Lee et al., 2003). Moreover, CRP encounters pathogenic bacteria and protects the tissues against further damage as well as it helps in tissue regeneration and repair (Kaya et al., 2016; Sarikaputi et al., 1992). Therefore, it could be used for evaluation the herd health status and might be benefit for early disease surveillance (Lee et al., 2003).

Diagnosis of SCE depends mainly on histological examination and counting of PMNs in endometrial cytology (Loyi et al., 2013; Nehru et al., 2019). However, these methods are not practical under field conditions due to the poor hygienic measures in rearing areas and bad temperament of buffaloes as well as the comparatively thin and folded genitalia (Srivastava and Kumaresan, 2014). Early possible prediction and diagnosis of uterine infections are essential for efficient pp. management (Manimaran et al., 2016). In this vein, the current study was designed to clarify the relation between SCE and postpartum ovarian resumption in Egyptian buffaloes. Moreover, the study aimed to evaluate serum TNF-α, IL-8 and CRP levels as well as endometrial TNF-α and IL-8 in buffaloes with and without SCE and their correlations with PMNs% in endometrial cytology.

## Materials and methods

### Animals and location

Pluriparous postpartum buffaloes in Educational Farm of Suez Canal University, Ismailia, Egypt were used. Buffaloes were examined regularly by farm veterinarians. All the used buffaloes were apparently healthy. They were free from mastitis, general health diseases and reproductive disorders. Before beginning of the study, buffaloes with clinical endometritis (n=7) were excluded after vaginoscopic examination and these animals were not used in the experiment.

Days in milk (DIM) of the previous lactation period was calculated for the studied animals. Buffaloes calved in winter and spring seasons. The mean parity of these buffaloes was 5.8 ± 0.2 (ranged from 4 to 8). Animals without clinical endometritis (n=39) were used throughout the study. Buffaloes were followed idealistic management practices and settled freely in an open yard. The study followed research ethical committee of the faculty guidelines (protocol # 2018056).

### Blood and endometrial sampling

Blood samples (n=117) were collected at the 3^rd^ (W3), 5^th^ (W5) and 7^th^ (W7) pp. weeks. They were collected from jugular vein in plain tubes and transported on ice to the laboratory. Sera were obtained by cooled centrifugation of blood samples at 3000 rpm for 15 minutes. The harvested sera were stored at -20 °C until analysis.

At W5 and W7 pp., duplicate of aseptic endometrial cytology were collected from each buffalo using autoclaved cytobrush (Puritan Medical products Co., Guilford, USA) according to Senosy and Hussein (2013). Cytobrushes were enclosed in sterilized plastic sheath. The enclosed cytobrush within specific device was introduced gently to the uterus then be rolled against endometrial wall. Later on, cytobrush was reinserted to the catheter before its withdrawal from uterus. The first one was smeared on glass microscope slide and fixed by cytofixative (Cytokeep ii, Alfresa pharma Co., Japan) according to Ahmed et al. (2017). The second one was directly immersed in cryovial and kept in liquid nitrogen for endometrial TNF-α and IL-8 analysis.

### Diagnosis of cytological endometritis and classification of buffaloes

Fixed endometrial cytological slides were transferred to the laboratory within an hour to be stained by Diff-Quik stain (Sysmex Co., Japan) according to the manufacturer’s instructions. Assessment of uterine cytology was performed in order to determine PMNs% via counting 200 cells using light microscope (X400). Based on the percentage of PMNs (threshold values of PMNs ≥ 6% at W5 or ≥ 4% at W7 pp.), buffaloes (n=39) were categorized into SCE and non-SCE groups (Ahmed et al., 2017; Ghanem et al., 2016; Senosy and Hussein, 2013).

### Endometrial sampling

The cytobrush was carefully weighed before collection of endometrial sample and after scratching of endometrium. The difference between the two weights was considered the actual weight of the endometrial scrape which was diluted by 10% (w/v) cold PBS (pH= 7.4) centrifuged at 3000 rpm for 20 minutes at 4 °C (Upreti et al., 1991). The supernatants were placed in eppendorf tubes and kept at -80 °C until analysis of TNF-α and IL-8.

### Resumption of ovarian cyclicity

Per rectal palpation, ultrasonography examination (Honda Electronics, Japan) as well as assessment of serum progesterone (P_4_) were performed at W3, W5 and W7 for monitoring of ovarian resumption. Serum P_4_ was assessed by bovine progesterone ELISA kit (Mybiosource Co., USA). Buffaloes with P_4_ levels ≥1.0 ng/mL were considered to have luteal activity (Palta and Madan, 1995; Usmani et al., 2001). While those with serum P_4_ levels ˂ 1.0 ng/mL were considered to be either in follicular phase or did not resume postpartum ovarian activity. Ovulation was considered to happen 5 days before the first outset of circulating P_4_≥ 1.0 ng/mL (Usmani et al., 2001). The animals that ovulate within ≤ 45 days postpartum, they considered to exhibit normal resumption of postpartum ovarian cyclicity. However, those of delayed ovarian resumption did not ovulate till > 45 days after calving (Ghanem et al., 2015; Shrestha et al., 2004).

### Pro-inflammatory cytokines and CRP

Bovine enzyme-linked immunosorbent assay (ELISA) kits were used for assessment of TNF-α and IL-8 (Cusabio Co., China,) at W5 and W7 in sera and endometrial samples. Briefly, pre-coated microplate with specific bovine antibody conjugates for either TNF-α or IL-8 were employed for competitive inhibition enzyme immunoassay technique. Both of standards and samples were added to the microtiter plate wells. A competitive inhibition reaction was launched with Horseradish Peroxidase (HRP) labeled target antigen and unlabeled antigen with the antibody. Substrate color development was inversely proportional to the amount of cytokines in the sample. The reaction was stopped and the color intensity was measured by ELISA reader (Das Co., Italy). Serum CRP was evaluated by using bovine CRP ELISA kit (Life Diagnostics, USA) at W5 and W7 according to the manufacturer’s guidelines.

### Statistical analysis

Differences in serum and endometrial levels of TNF-α, IL-8 and serum CRP between SCE and non-SCE groups at W5 and W7 were assessed by Mann-Whitney nonparametric analysis. The PMNs% and parity of SCE subgroups and non-SCE buffaloes were analyzed by nonparametric Kruskal-Wallis test. Further comparisons among different groups were done by Dunn’s test for multiple comparisons. The prevalence of normal and delayed ovarian cyclicity at W7 pp. in buffaloes with and without subclinical endometritis was analyzed by Fisher’s exact test. The correlation coefficient between serum and endometrial TNF-α, IL-8 and serum CRP and their correlation with PMNs% were analyzed by Spearman’s test (GraphPad Prism software version 7.0, San Diego, USA). Logistic regression analysis (IBM SPSS software computer program version 20, USA) was applied to evaluate the effect of calving season, serum P_4_ levels in W3, W5 and W7 as well as DIM on PMNs% (as indicator for SCE).The obtained data are presented as means with SEM. Significance was designated at probability value of *P* < 0.05.

## Results

### Grouping, parity, PMNs% and ovarian status of buffaloes at W5 and W7

According to PMNs %, buffaloes (n=39) were divided into SCE group (n=27) and non-SCE group (n=12). Twelve out of 39 buffaloes did not exhibit SCE neither at W5 nor W7, while 3 exhibited SCE at W5 not W7, six exhibited SCE at W7 not W5 and the remaining 18 exhibited SCE at both W5 and W7 (Table 1).

**Table 1:** Grouping, parity, PMNs%, and ovarian activity of buffaloes without and with subclinical endometritis (SCE).

**Groups**	**Buffaloes No.**	**Parity**	**PMNs %**		**Buffaloes resumed luteal activity** **No. (%)**
			**W5**	**W7**	**W7**
**Non-SCE Buffaloes**	12	6.3±0.4	2.5±0.5^a^	0.8±0.1^a^	10 (83.3%)
SCE Buffaloes:					
SCE W5	3	5.7±0.9	10.0±0.6^b^	2.0±0.6^a^	2 (66.7%)
SCE W7	6	5.7±0.5	1.7±0.4^a^	10.0±1.3^b^	3 (50.0%)
SCE W5 and W7	18	5.6±0.3	10.8±2.1^b^	8.7±1.7^b^	7 (38.9%)
Total	39				22 (56.4%)
*P* value		0.640	˂0.001	<0.001	

Means with different superscripts within the same column are significantly different at *P* < 0.001. *P:* probability.

No significant differences (*P* > 0.05) were recorded in parity between the two groups of buffaloes. At W5, SCE W5 group as well as SCE W5 and W7 group showed significant (*P*< 0.001) elevation in PMNs% as compared to non-SCE and SCE W7 groups. While at W7, SCE W7 and both SCE W5 and W7 groups showed significant (*P* < 0.001) increase in PMNs% compared to that in those without SCE and SCE W5 buffaloes (Table 1).

Based on serum P_4_ concentration (≥ 1.0 ng/mL) in buffaloes; 10 (83.3%) out of 12 non-SCE buffaloes, 2 (66.7%) out of 3 SCE buffaloes at W5, 3 (50.0%) out of 6 SCE buffaloes at W7 and 7 (38.9%) out of 18 SCE buffaloes at W5 and W7 were assumed to resume the luteal activity (Table 1).

### Postpartum resumption of ovarian cyclicity in SCE and non-SCE buffaloes

Whereas normal pp. ovulation happens ≤ 45 days after calving, SCE buffaloes manifested a significant (*P* = 0.036) delay in resumption of pp. luteal activity compared with that in non-SCE buffaloes at W7 (Table 2).

**Table 2:** Normal and delayed ovarian cyclicity of SCE and non-SCE buffaloes at the end of postpartum period.

**Buffalo Groups**	**No. (%)**	**Normal ovarian cyclicity** **No. (%)**	**Delayed ovarian cyclicity** **No. (%)**	***P* value**
SCE	27 (69.2%)	12 (44.4%)	15 (55.6%)	0.036
Non-SCE	12 (30.8%)	10 (83.3%)	2 (16.7%)	

*P:* probability.

### Levels of cytokines and CRP and their correlations

Results showed significant elevations in serum TNF-α (*P* = 0.023) and IL-8 (*P* = 0.036) in SCE buffaloes when compared to that in non-SCE group at W5. The same trend was observed (*P*= 0.009) in all parameters at W7 (Table 3). Buffaloes with SCE showed significant increments in endometrial TNF-α and IL-8 at W5 (*P* < 0.001) and at W7 (*P* < 0.01) compared to non-SCE buffaloes (Table 3).

**Table 3:** Serum and endometrial levels of TNF-α and IL-8 in non-SCE and SCE buffaloes and their correlations at W5 and W7.

	**Serum**						**Endometrium**						**Correlations**			
	**W5**		**P**	**W7**		**P**	**W5**		**p**	**W7**		**p**	**W5**		**W7**	
	**Non- SCE**	**SCE**		**Non- SCE**	**SCE**		**Non-SCE**	**SCE**		**Non-SCE**	**SCE**		**r**	**P**	**r**	***P***
**TNF-α** (pg/mL)	62.67±3.4	314.3±20.8	0.023	71.3±9.4	347.7±14.5	0.009	440±7.3	652.5±17.4	0.0007	470±20.3	581.7±25.3	0.006	0.76	0.05	0.69	0.05
**IL-8** (pg/mL)	49.2±18.1	148.5±15.3	0.036	31.38±4.6	188.1±11.5	0.009	840.3±4.2	1026±16.8	0.0007	961±8.7	1115±35.5	0.002	0.89	0.01	0.92	0.001

TNF-α: Tumor necrosis factor alpha; IL-8: Interleukine-8; *P:* probability; r: correlation coefficient.

**Figure 1:**
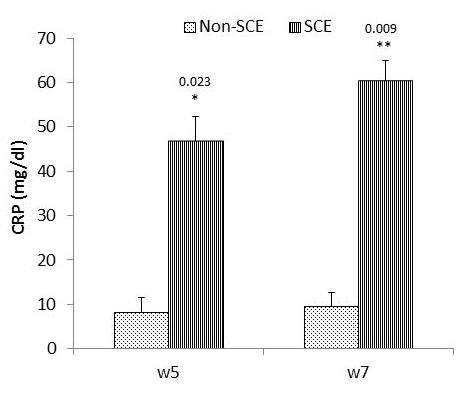
Serum levels of CRP (mg/dl) in SCE and non-SCE buffaloes at W5 and W7. *Significantly different (*P* <0.05). **Highly significantly different (*P* <0.01).

Serum CRP levels revealed significant (*P* = 0.023 and 0.009) elevations in SCE group as compared to non-SCE group at W5 and W7, respectively (Figure 1).

Serum TNF-α and IL-8 were positively correlated to their respective endometrial levels at W5 (*P* < 0.05 and 0.01, respectively) and at W7 (*P* < 0.05 and 0.001, respectively) as illustrated in Table 3.

### Correlations between PMNs%, cytokines and CRP in buffaloes

In non-SCE group, the PMNs% revealed non-significant correlations (*P* > 0.05) with serum and endometrial TNF-α, IL-8 and serum CRP levels at W5 and W7. While, PMNs% of SCE group at W5 showed significant correlations with both serum and endometrial levels of TNF-α (*P*< 0.05), IL-8 (*P* < 0.01) and serum CRP (*P* < 0.05). Moreover, SCE buffaloes revealed significant correlations in cytological PMN% at W7 with serum and endometrium TNF-α (*P*< 0. 01 and < 0.05, respectively) and IL-8 (*P* < 0.001 and *P* < 0.05, respectively) as well as serum CRP (*P* < 0.01) (Table 4).

**Table 4:** Correlation coefficients between PMNs%, serum and endometrial levels of TNF- α, IL-8 and CRP of non-SCE and SCE buffaloes at W5 and W7 postpartum.

**Groups**	**W5**				**W7**			
	**Serum**		**Endometrium**		**Serum**		**Endometrium**	
	**r**	***P***	**r**	***P***	**r**	***P***	**r**	***P***
**Non-SCE group**								
PMNs% and TNF-α	-0.15	ns	-0.15	ns	0.63	ns	0.07	ns
PMNs% and IL-8	-0.43	ns	-0.43	ns	0.69	ns	0.66	ns
PMNs% and CRP	-0.50	ns			0.42	ns		
								
**SCE group**								
PMNs% and TNF-α	0.87	0.05	0.86	0.05	0.93	0.01	0.77	0.05
PMNs% and IL-8	0.40	0.01	0.82	0.01	0.80	0.001	0.81	0.05
PMNs% and CRP	0.65	0.05			0.61	0.01		

PMNs%: Polymorphonuclear cells %; TNF-α: Tumor necrosis factor alpha; IL-8: Interleukine-8; CRP: C-reactive protein; *P:* probability; r: correlation coefficient; ns: non-significant.

### Effects of calving season, serum P_4_ levels and DIM on PMNs%

Logistic regression analysis indicated non-significant effects of calving season, serum P_4_ levels at W3, W5 and W7 as well as DIM on PMNs% (Table 5).

**Table 5:** Logistic regression analysis of the effect of calving season, serum progesterone levels at W3, W5 and W7 as well as DIM on polymorphonuclear cells % (PMNs%).

**parameters**	**β**	**Wald statistics**	***P* value**
**Season**	-.167	.015	.902
**P_4_ at W3**	-1.849	3.226	.072
**P_4_ at W5**	-3.032	2.065	.151
**P_4_ at W7**	-1.842	1.204	.273
**DIM**	.005	.006	.937

DIM: Days in milk; β: regression coefficient; *P:* probability.

## Discussion

The postpartum period is critically substantial for fertility and reproductive performance of buffaloes. Uterine diseases especially SCE is associated with lower productive and reproductive performances (Senosy and Hussein, 2013). The uterine natural resistance mechanisms usually terminate uterine infection and inflammation. However, infection persists in some animals and causes subfertility even after resolution of clinical signs (Kasimanickam et al., 2004, 2005).

A complex relationship exists among factors including the immune system that influencing uterine health and disease condition in postpartum period. Therefore, the current study was planned to validate the use of serum TNF-α, IL-8 and CRP as diagnostic alternate for their uterine levels for SCE in clinically normal postpartum buffaloes. Moreover, the study evaluated the impact of SCE on resumption of postpartum ovarian cyclicity.

The high incidence of SCE (69.2 %) that observed in our study could be attributed to the high liability of buffaloes to uterine infections due to the poor hygienic conditions in the rearing areas. Moreover, buffaloes behave improper practices as vaginal stimulation for milk let-down as well as their wallowing habit (Azawi et al., 2008).

Some buffaloes were positive for SCE at W5 but not at W7. This might be explained by the capability of uterus to eliminate the infection. On the other side, the buffaloes that were negative to SCE at W5 but acquired it at W7. This could be attributed to the lowered immune response of endometrium postpartum that was well consolidated in cattle as recorded by Ghanem et al. (2015). The same findings were recorded by Knutti et al. (2000) who found that the untreated cows suffered from mild endometritis had high self-healing ability of the uterus.

The delay of postpartum resumption of ovarian cyclicity in SCE group was in accordance with Pande et al. (2013) who found that buffaloes suffered from cytological endometritis had lower levels of circulating P_4_ that adversely influence the resumption of ovarian cyclicity. Genital infections had deleterious effects on bovines' reproductive pattern through suppressing secretion of pituitary LH as well as outfaces postpartum ovulation (Hanafi et al., 2008; Manimaran et al., 2016; Williams et al., 2005). Our study confirmed the existence of relations between the elevated endometrial and serum levels of TNF-α, associated IL-8 and serum CRP with the reduced levels of circulating P_4_ in SCE buffaloes. Endometrial cytokines which produced during uterine inflammation could obscure ovulation due to low concentrations of plasma estradiol and slow growth of the dominant follicle as well as reducing P_4_ secretion (Sheldon et al., 2009). Moreover, the levels of TNF-α and some cytokines such as IL-8 play a modulating role inside bovine corpus luteum steroidogenic cells (Korzekwa et al., 2006; Taniguchi et al., 2002) to enhance luteolytic action of PGF2α (Skarzynski et al., 2005) and endothelin-1 (Ohtani et al., 1998) thus inhibited the local release of P_4_ and hence gonadotropin supported P_4_ production. This led to delayed resumption of ovarian activity in SCE buffaloes where uterine and serum TNF-α were elevated (Korzekwa et al., 2008). Actually, the level of TNF-α is crucial for ovarian function where different studies suggested that adequate physiological levels of TNF-α could support ovarian function and cyclicity. However, higher abnormal levels as observed here in SCE buffaloes increase caspase-3 activity of bovine luteal cells that subsequently decrease P_4_ production (Korzekwa et al., 2008). On the other hand, Usmani et al. (2001) noted that subclinical uterine infections did not influence the onset of ovarian cyclicity in postpartum buffaloes.

After parturition, the physiological barrier of the genital system becomes loosen and the pathogenic opportunistic microorganisms invade the uterus. The endometrial immunity responds to bacterial agents via production of pro-inflammatory cytokines (Fischer et al., 2010; Patra et al., 2014) which control the uterine infection through stimulation of immune cell mobilization into the uterus (Patra et al., 2014). Moreover, the emission of uterus pro-inflammatory cytokines is magnified by their paracrine actions that results in further production and eventually systemic increase in cytokines (Stassi et al., 2017). The elevated serum and endometrial TNF-α in SCE buffaloes at W5 and W7 postpartum was in accordance to Kasimanickam et al. (2013) and Brodzki et al. (2015) in cows, Fischer et al. (2010) and Singh (2017) in buffaloes. Levels of TNF-α was considered a key cytokine which involved in adhesion molecules, like E-Selectin and IL-8 expression that play an important role in PMNs recruitment (Bhadaniya et al., 2019; Roach et al., 2002). On the parallel line, serum and endometrial IL-8 levels were promoted in SCE group.

The observed elevation in IL-8 levels augmented the elevation of TNF-α that induced its production and further uterine PMNs chemotaxis (Manimaran et al., 2016; Nehru et al., 2019). Similar findings were obtained by Mossallam et al. (2015) who recorded significant increase in the IL-8 levels in uterine tissue of SCE buffaloes. Furthermore, Singh (2017) observed increments in both circulating and endometrial IL-8 levels in SCE buffaloes when compared to control group. The possible explanation of the synergistic elevation of both TNF-α and IL-8 levels is that the endometrial cells respond to infection by activation of TLRs (Ajevar et al., 2014) and thereafter stimulate the release of pro-inflammatory cytokines as TNF-α, chemokines as IL-8 and prostaglandins (Fischer et al., 2010; Loyi et al., 2013). However, Kim et al. (2014) and Galvão et al. (2012) reported no differences in serum levels of TNF-α and IL-8 of SCE cows compared with control animals. The increase in TNF-α and IL-8 concentrations in SCE buffaloes was due to remarkable synthesis of endometrial immune cells as well as peripheral blood cells in response to SCE (Brodzki et al., 2015).

The congruent positive correlation between PMNs% and serum as well as endometrial levels of TNF-α and IL-8 in SCE buffaloes invigorated the permeation of neutrophils into uterus for withdrawal of the pathogenic micro-organisms (Kasimanickam et al., 2004; Warren, 1990). This could be attributed to the observed promotion in TNF-α and subsequently IL-8 that chemoattracts PMNs into the uterus (Patra et al., 2014) and hasten the endometrial infiltration of PMNs (Bhadaniya et al., 2019; Fischer et al., 2010).

C-reactive protein is an essential element of innate system defense that regulates the inflammatory response and acts as a safeguard against infection (Sarikaputi et al., 1992). Moreover, it reacts with receptors on phagocytic cells to mediate phagocytosis and motivate anti-inflammatory cytokines production (Du Clos and Mold, 2001). The elevations in the circulating CRP of SCE buffaloes at W5 and W7 as well as their positive correlation with PMNs% was in line with Li et al. (2010) and Kaya et al. (2016) who recorded significant elevations in serum CRP levels of clinical and subclinical endometeritic cows when compared to healthy animals. The serum CRP levels increased by about 6 folds in SCE buffaloes than control that could be attributed to the promotion of mononuclear cells in response to uterine infection to produce their cytokines TNF-α and IL-8 which subsequently stimulated the emission of CRP from liver into blood stream (Kaya et al., 2016; Lee et al., 2003). The significant elevation of serum CRP in buffaloes with SCE may indicate an important involvement of CRP as diagnostic key in SCE. Therefore, assessments of serum CRP besides TNF-α and IL-8 could be potent predictors for uterine inflammation in apparently healthy bovine (Kaya et al., 2016; Loyi et al., 2013) especially when the endometrial sampling is difficult to be obtained.

In conclusion, SCE was implicated in delay of postpartum ovarian resumption in Egyptian buffaloes. The elevated concentrations of endometrial TNF-α and IL-8 could be reflected on their serum levels that might have a diagnostic importance for SCE in Egyptian buffaloes. Hence, the complications met by field conditions to obtain endometrial sample for SCE diagnosis, the serum levels of TNF-α, IL-8 and CRP could introduce a logic alternate to diagnose SCE in buffaloes.
